# Tc17/IL-17A Up-Regulated the Expression of MMP-9 via NF-κB Pathway in Nasal Epithelial Cells of Patients With Chronic Rhinosinusitis

**DOI:** 10.3389/fimmu.2018.02121

**Published:** 2018-09-19

**Authors:** Xiaohong Chen, Lihong Chang, Xia Li, Jiancong Huang, Luoying Yang, Xiaoping Lai, Zizhen Huang, Zhiyuan Wang, Xifu Wu, Jun Zhao, Joseph A. Bellanti, Song Guo Zheng, Gehua Zhang

**Affiliations:** ^1^Department of Otorhinolaryngology, The Third Affiliated Hospital of Sun Yat-Sen University, Guangzhou, China; ^2^Department of Clinical Immunology, The Third Affiliated Hospital of Sun Yat-Sen University, Guangzhou, China; ^3^Department of Pediatrics and Microbiology-Immunology, Georgetown University Medical Center, Washington, DC, United States; ^4^Division of Rheumatology, Milton S. Hershey Medical Center at Penn State University, Hershey, PA, United States

**Keywords:** chronic rhinosinusitis, IL-17A, Tc cells, nasal epithelial cells, MMP-9, NF-κB

## Abstract

Chronic rhinosinusitis (CRS) is a common chronic inflammatory disease of the upper airways involving nasal cavity and sinus. Deriving both from its clinical complexity with protean clinical manifestations as well its pathogenetic heterogeneity, the molecular mechanisms contributing to the pathogenesis of CRS remain unclear, and attract a wide interest in the field. Current evidences indicate that IL-17A is highly expressed in chronic rhinosinusitis with nasal polyps (CRSwNP). However, its pathogenetic role in regulation of tissue remodeling of CRSwNP remains unknown. The present study aimed to investigate the cellular origins and functions of IL-17A cytokine in CRSwNP, and further determined whether IL-17A could affect the expression of metalloproteinases (MMPs), the remodeling factors of CRSwNP. The results showed that the expression of IL-17A was upregulated in nasal tissues of patients with CRSwNP compared to those with chronic rhinosinusitis without nasal polyps (CRSsNP) and controls. CD8^+^ cytotoxic T lymphocytes (Tc) were major IL-17A producers in nasal tissues of CRSwNP. Interleukin (IL)-17-producing CD8^+^ T cells (Tc17) was significantly higher in nasal tissues of CRSwNP than CRSsNP and controls. Nonetheless, no difference was observed among the IL-17A in peripheral blood lymphocytes of these three groups. Moreover, in the same patients, IL-17A expression was negligible in lymphocytes of peripheral blood when compared with nasal tissues. Increased gene and protein expression of MMP-7 and MMP-9 in patients with CRSwNP compared with controls were observed. In CRSwNP samples, IL-17A receptor (IL-17AR) co-localized with MMP-9 and they were mainly expressed in the epithelial cells. MMP-9 expression was up-regulated both in Primary human nasal epithelial cells (PHNECs) and a nasal epithelial cell line (RPMI 2650) by IL-17A treatment, and diminished by anti-IL-17AR treatment. Furthermore, IL-17A promoted the expression of MMP-9 by activating the NF-κB signal pathway. Thus, our results have revealed a crucial role of IL-17A and Tc cells on pathogenesis and tissue remodeling of CRSwNP.

## Introduction

Chronic rhinosinusitis (CRS) is a common chronic inflammatory disease of the upper airways involving nasal cavity and sinus, with an estimated worldwide prevalence of 5–16% (1–3). As defined in the European Position Paper on Rhinosinusitis and Nasal Polyps 2012 (EPOS2012) of the European Rhinologic Society, CRS is currently divided on the basis of distinct tissue remodeling patterns into CRS with nasal polyps (CRSwNP) and CRS without nasal polyps (CRSsNP) (4). In contrast to CRSwNP which is typically characterized by excessive degradation of the extracellular matrix (ECM) and the presence of pseudocyst formations with albumin accumulation and edema formation (5), CRSsNP is defined by excessive extracellular matrix (ECM) deposition and fibrosis formation (6). Matrix metalloproteinases (MMPs), a family of Ca^2+^-activated, zinc-dependent endopeptidases, play a key role in the degradation of basement membranes and extracellular matrix (ECM) (7, 8). Recent studies have suggested that the imbalance between MMPs and their tissue inhibitor of metalloproteinases (TIMPs) is one of the most causes leading to the pathological tissue remodeling seen frequently in CRS (9–11). However, the detailed upstream regulation of MMPs/TIMPs balance that promotes the tissue remodeling remains largely unclear.

In addition to tissue remodeling patterns, CRSwNP and CRSsNP possess distinct immunophathlogical characteristics. European studies showed that CRSwNPs is characterized by a Th2-skewed pattern of eosinophalic inflammation whereas in CRSsNP a predominant Th1 milieu is seen (12, 13). Interestingly, CRSwNP in Asian patients present different characteristics. Zhang et al. found that CRSwNP patients from Southern China showed a Th1/Th17 cell pattern with minor eosinophilic inflammation (14, 15). In contrast, in studies of Chinese patients with an eosinophilic pattern of CRSwNPs by Cao et al. a predominant Th2/Th17 cell function response was seen but not in patients with the non-eosinophilic form Cao et al. (16). Therefore, further understanding of the role of IL-17 in CRS will be of great value in exploring the pathogenesis of this disorder.

IL-17A, commonly referred to as IL-17, is a member of the IL-17 cytokine family and was the first identified and, by far, the best studied (17). It has been associated with the pathology of numerous autoimmune and inflammatory diseases such as asthma (18–20), rheumatoid arthritis (21–24), allergic rhinitis (25), acute hepatic injury (26, 27), periodontitis (28). In these disorders, IL-17 is known to increase the production of proinflammatory cytokines, chemokines, and MMPs (29). IL-17A has also been reported to promote the invasion of cancer cells by inflammatory cells by upregulating the expression of MMP-2 and MMP-9 (30, 31). Interestingly, although previous studies have consistently shown that IL-17A is highly expressed in CRSwNP (32, 33), its pathogenetic role in regulation of extracellular matrix balance in CRS remains unknown.

The purpose of the present study was to explore the cellular origin and clinical significance of IL-17A in CRSwNP, as well as to determine its possible molecular mechanistic role in regulating tissue remodeling.

## Materials and methods

### Patients and tissue samples

This study was approved by the Ethics Committee of the Third Affiliated Hospital of Sun Yat-sen University (No.[2013]2-9) and all enrolled subjects had given their written informed consent. In the study, 55 patients with CRSsNP patients, 132 CRSwNP patients, and 50 control subjects were enrolled. The diagnosis of CRS was based on the guidelines of EPOS 2012 (4). Control subjects consisted of patients who underwent non-sinonasal-related surgeries, such as repair of simple nasal septum deviation, cerebrospinal fluid rhinorrhea, and pituitary tumor. Nasal polyps and ostiomeatal complex (OMC) mucosa from CRS patients and inferior turbinate mucosa from control subjects were obtained during surgeries. Only adult subjects ranging in age from 18 to 65 year-old without antibiotic, corticosteroid or other immune-modulating drug therapy up to 4 weeks before surgery were included in the study. Subjects who had autoimmune diseases, aspirin intolerance triad, unilateral rhinosinusitis, antrochoanal polyps, allergic fungal rhinosinusitis, cystic fibrosis, or immotile ciliary disease were excluded. Due to the limited amounts of tissues available on biopsy, not all samples were included in each study method. Nasal biopsies were immediately frozen and stored in liquid nitrogen or−80°C for subsequent isolation of mRNA and proteins, or for histologic examination of frozen slice sections. Partial nasal biopsies from CRSwNP were freshly treated to isolate nasal epithelial cells for cell culture as previously described (34), and other nasal biopsies were freshly processed to isolate single cell suspensions to analysis cytokines and other inflammatory mediators by flow cytometry. The atopic status of the subjects was evaluated with one venous blood sampling for determination of IgE antibodies against common aeroallergens (house dust mites, molds, trees, weeds, grass, and animal dander, etc.). A diagnosis of asthma was performed by an allergist based on medical history and lung function analysis. More detailed descriptions of subjects characteristics are provided in Table 1.

**Table 1:** Characteristics and methods.

	**Control**	**CRSsNP**	**CRSwNP**
Total subject number	50	55	132
Gender, male/female	37/13	38/17	97/35
Age(y), mean (*SD*)	33 (11)	40 (14)	40 (15)
Atopy, *N* (%)	18 (36.00)	15 (27.27)	56 (42.42)
Asthma, *N* (%)	0	0	5 (3.79)
Aspirin sensitivity, *N* (%)	0	0	0
Smoking, *N* (%)	3 (0.06)	6 (10.91)	19 (14.39)
**METHODOLOGIES USED**			
Flow cytometry	14	22	52
Homogenate ELISA	9	9	12
Tissue mRNA	26	26	58
Cell culture	0	0	9
Immunflourescence	1	1	6

### Human nasal epithelial cells (HNECs) culture

Primary human nasal epithelial cells (PHNECs) were prepared from specimens obtained from individual subjects. Nasal polyps of CRSwNP group were washed thoroughly and digested in 2 mg/ml protease (type XIV, Sigma-Aldrich, St Louis, MO, USA) in Dulbecco's Modified Eagle's Medium (DMEM, Thermo Scientific Inc., New York, USA) overnight at 4°C. After digestion, epithelial cells were released by vigorous shaking. Since the hybrid fibroblasts were preferentially adherent, impure epithelial cells were placed on a plastic dish at 37°C for 1 h to eliminate fibroblasts. High purity epithelial cells were then collected and cultured through a filter screen in bronchial epithelial growth medium (BEGM, Lonza, Basel, Switzerland) at a density of 5 × 10^5^ cells/cm^2^ at 37°C in an atmosphere of 5% CO_2_ and 95% relative humidity. RPMI 2650 (Sigma-Aldrich), a nasal epithelial cell line, was used as a source of normal nasal mucosal epithelial cells as a methodologic cell control and cultured in 1640 (Thermo Scientific) with 10% Fetal Bovine Serum (FBS, Biowest, Loire Valley, France) at 37°C in an atmosphere of 5% CO_2_ and 95% relative humidity (35).

### Quantitative real-time RT-PCR

The mRNA expression levels of MMPs (MMP-2,7,9) in tissue samples from controls, CRSsNP and CRSwNP and in isolated cells culture samples were analyzed in differentially-treated specimens. Total RNA was extracted from tissues and cells by RNAiso Plus (TaKaRa Biotechnology, Dalian, China). One microgram of total RNA was reverse-transcribed to cDNA with a PrimeScript RT reagent kit (TaKaRa Biotechnology). Quantitative real-time PCR was performed by using the SYBR Premix Ex Taq kit (TaKaRa Biotechnology) and the appropriate primers (Invitrogen, Carlsbad, CA, USA) were presented in Table 2. Expression of β2 microglobulin (β2M) was served as a housekeeping gene for normalization. Relative gene expression was carried out with comparative 2^−ΔΔ*CT*^ method (36, 37). To analyze the data, we used Sequence Detection Software (version 1.9.1, Applied Biosystems).

**Table 2:** Primers used for real-time PCR analysis.

**Target**	**Primer sequence**
MMP-2	(F)5′-CCTCCCGGTGCCCAAGAATAGA-3′
	(R)5′-GGCTCTGAGGGTTGGTGGGATT-3′
MMP-7	(F)5′-GAGGCATGAGTGAGCTACAGTG-3′
	(R)5′-CATCTCCTTGAGTTTGGTTCT-3′
MMP-9	(F)5′-AGACCTGGGCATTCCAAAC-3′
	(R)5′-CGGCAAGTCTTCCGAGTAGT-3′
β2M	(F)5′-TACACTGAATTCACCCCCAC-3′
	(R)5′-CATCCAATCCAAATGCGGCA-3′

*MMP, matrix metalloproteinase; β2M, β2 microglobulin; reference gene*.

### Western blot analysis

PHNECs and RPMI 2650 were preincubated with Recombinant Human IL-17A (Peprotech, New Jersey, USA)/anti-IL-17AR (R&D systems, Minneapolis, MN, USA)/BAY 11-7082 [MedChemExpress (MCE), New Jersey, USA]/DMSO (MP Biomedicals, California, USA) and the expression of MMPs and NF-κB were analyzed by Western blotting. Total proteins were extracted from cells (PHNECs and RPMI 2650) by loading buffer (Thermo Scientific Inc., New York, USA) and electrophoresed on sodium dodecyl sulfate-polyacrylamide gel electrophoresis (SDS-PAGE, Sigma-Aldrich), and transferred to polyvinyl difluoride membranes (PVDF, Merck Millipore, Darmstadt, Germany). Membranes were blocked with 5% Albumin Bovine V (Biotopped Science & Technology, Beijing, China) in Tris-buffered saline (Boster Biotechnology, Wuhan, China) with Tween-20 (Solarbio Science & Technology Company, Beijing, China) for 1 h at room temperature and incubated overnight at 4°C with mouse anti-human MMP-9 mAb (Abcam, Cambridge, UK), anti-human MMP-7 mAb (Cell Signaling, Danvers, MA, USA) mouse Anti-human P-IκBα antibody (Cell Signaling), mouse Anti-human IκBα antibody antibody (Cell Signaling), or GAPDH antibodies (proteintech, Chicago, United States). After wahed with TBST, the membranes were incubated with horseradish peroxidase (HRP)-conjugated rabbit/mouse anti-goat IgG (Bioworld Technology, Minnesota, USA) for 90 min at room temperature and visualized by using the ECL reaction (advansta, California, USA) with Image Reader. GAPDH quantification was used as an internal standard to correct the total protein loading.

### ELISA

Tissue samples were mortar-ground with liquid nitrogen, following which each resultant tissue dry powder specimen was then weighed, and 100 μL PBS supplemented with 10% protease inhibitor cocktail (MedChem Express, Shanghai, China) was added per 10 mg tissue. After homogenization, the supernatants were separated and prepared. Tissue homogenates and cytokine concentrations in supernatants of cultured PHNECs were measured by commercial ELISA kits (CUSABIO, Wuhan, China) (38). All protocols were carried out according to the manufacture's recommendations. All the protein levels were normalized to total protein concentrations.

### Flow cytometry

Nasal mucosal cells (NMCs) and peripheral blood mononuclear cells (PBMCs) were isolated from nasal tissues and blood, respectively. Fresh nasal tissues were washed and cut into small pieces and then were prepared by enzymatic digestion of tissue fragments with trypsin (Invitrogen). Single cell suspensions were then obtained by grinding the tissues through a cell strainer of 40 μm. Purified suspensions of PBMCs were obtained by density gradient centrifugation of heparinized whole blood specimens using Lymphoprep (Alere Technologies AS, Oslo, Norway). The PBMCs in each sample were adjusted to a concentration of 2 × 10^6^ cells/mL following which 1 mL aliquots of cell suspensions were stimulated with a 2 μL cell activate ion cocktail (with Brefeldin A) (Biolegend, San Diego, CA, USA) for 5–6 h. Prior to flow cytometry, each sample was divided into three equal parts and subjected to three different protocols involving direct staining with specific conjugated antibody preparations prior to Six, Seven, eight-color flow cytometry, respectively. For flow cytometry, cells were first stained with the Zombie UV^TM^ Fixable Viability Stain (Biolegend) to exclude dead cells. Cells were surface labeled for 30 min at 4°C. The first protocol includes CD3 APC, CD8a APC/FireTM750, TCRαβ PE/Cy7, TCRγδ PE; The second protocol includes CD3 APC, CD19 APC/FireTM750, CD56 PE/Cy7, CD45 PerCP/Cy5.5; The third protocol includes CD8 APC/FireTM750, TCRαβ PE/Cy7, TCRγδ PE. For intracellular staining, following cell surface staining, cells were next prepared for intracellular staining in three protocols. Cells were fixed and permeablized using Fixation Buffer (Biolegend) and Intracellular Staining Perm Wash Buffer (Biolegend) under the manufacturer's protocols, and then stained for 30 min at 4°C. The first protocol includes IL-17A PE/DazzleTM 594, CD4 PerCP/Cy5.5; the second protocol includes IL-17A PE/DazzleTM 594; the third protocol includes IL-17A PE/DazzleTM 594, CD4 PerCP/Cy5.5, IL-4 Alexa Fluor@647, IFN-γ Alexa Fluor@488. All antibodies were purchased from Biolegend. Flow cytometric analysis was conducted with a flow cytometer (BD LSRFortessa^TM^, New Jersey, USA). Data were analyzed using FlowJo software (TreeStar, Ashland, OR, USA).

### Immunofluorescence

Immunofluorescence staining was performed on nasal tissue from CRSwNP to evaluate the co-localization of IL-17AR and MMP-9. Immunostaining was carried out using goat anti-human IL-17AR polyclonal antibody (Abcam) and mouse anti-human monoclonal MMP-9 antibody (Abcam). Double labeling was performed by secondary fluorescein isothiocyanate-conjugated donkey anti-goat antibodyand phycoerythrin (PE)-conjugated goat anti-mouse antibody (Sigma-Aldrich) in the same sections. Nasal epithelial cells (HNECs) were pretreated with IL-17A and were incubated by MMP-9 antibody (mouse monoclonal; 1:200; Abcam) and Cy3-labeled goat anti-mouse IgG antibody (1:100; invitrogen), respectively. Slides were visualized by a fluorescence microscope (Leica).

### Statistical analysis

Statistical analysis were performed using IBM SPSS 20 (SPSS, Chicago, Ill) and GraphPad Prism 7.0 (GraphPadSoftware, La Jolla, Calif) software. Data were presented as means and standard error of the mean (mean ± SEM). If not normally distributed, data were expressed as median (25–75 percentiles). Horizontal bars represent median, and error bars show interquartile range. For statistical analysis, Kruskal-Wallis tests were used to assess significant intergroup variability. Mann-Whitney *U*-tests (2-tailed) or Student *t*-tests were used for between-group comparisons for unpaired data. Paired Student *t*-tests or Wilcoxon matched-paired signed rank test were performed where applicable. *P* < 0.05 was accepted as statistically significance.

## Results

### The expression of IL-17A in CRS

**Figure 1:**
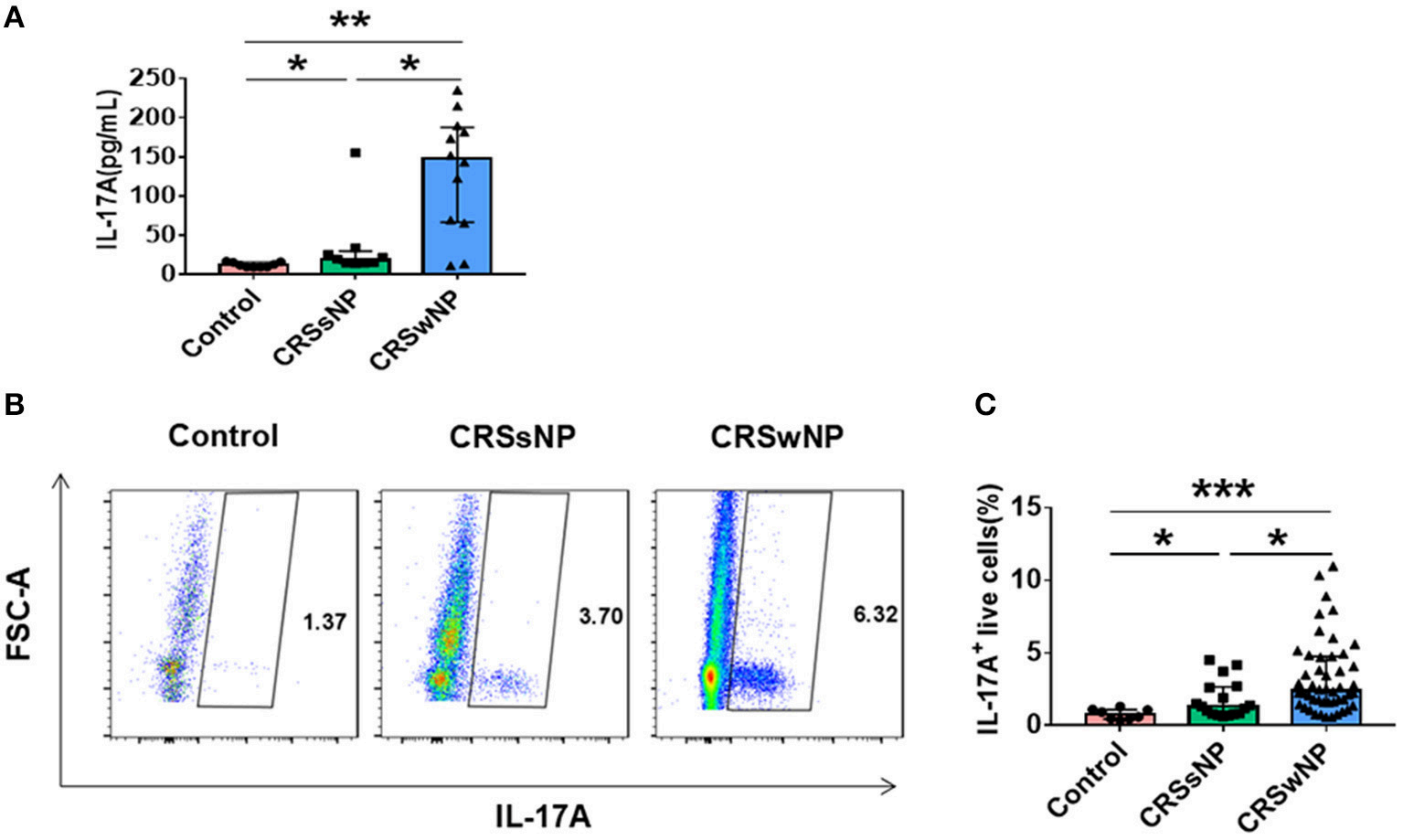
Expression of IL-17A in CRSsNP, CRSwNP patients, and control. **(A)** Concentration of IL-17A in the supernatants was assayed by ELISA (Control = 9, CRSsNP = 9, CRSwNP = 12). **(B)** Detection of IL-17A-producing live cells by flow cytometry (Control = 8, CRSsNP = 17, CRSwNP = 48). ^*^P < 0.05; ^**^P < 0.01; ^***^P < 0.001; ns, *P* > 0.05.

Tissues obtained from patients with CRSsNP, CRSwNP, and control subjects analyzed for IL-17A expression by ELISA demonstrated that IL-17A protein levels were significantly increased in patients with CRSwNP and CRSsNP compared with controls (*P* = 0.001, *P* = 0.012). IL-17A protein levels were also higher in patients with CRSwNP in comparison with CRSsNP. (*P* = 0.028) (Figure 1A). Concordant with the ELISA findings, flow cytometric analysis revealed an increased percentage of IL-17A^+^ live cells in both CRSwNP and CRSsNP compared with controls (*P* < 0.001, *P* = 0.02). Higher IL-17A^+^ levels were observed in CRSwNP than in CRSsNP cells (*P* = 0.011) (Figure 1B). Collectively, our data showed that patients with CRSwNP possessed significantly increased IL-17A expression.

### Tc cells (CD3^+^CD8^+^) are major IL-17A producers in nasal tissues from CRSwNP

**Figure 2:**
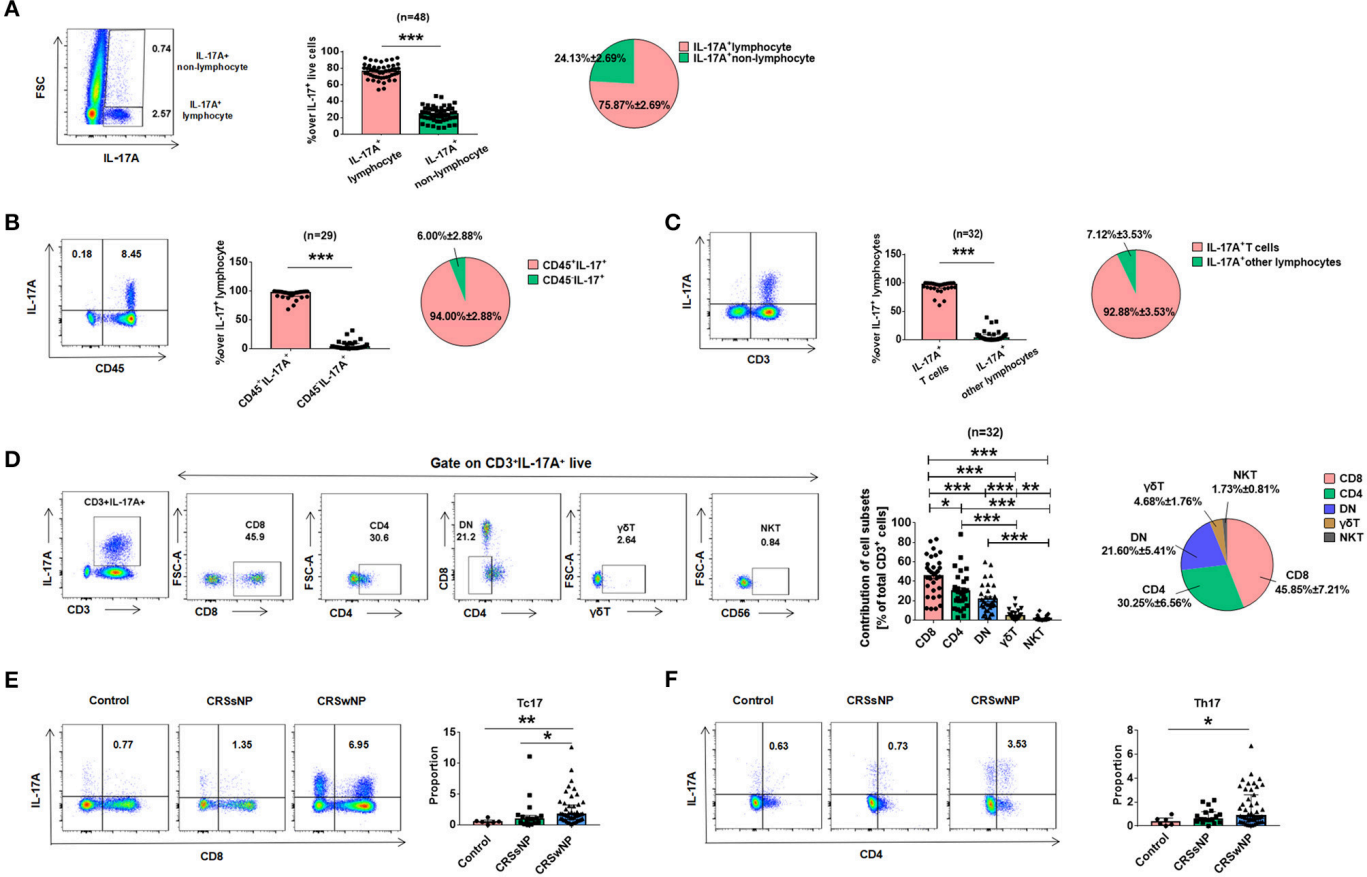
The cellular provenance of IL-17A in CRSwNP. **(A)** IL-17A was mainly derived from lymphocytes rather than other cells. **(B)** IL-17A was mainly derived from CD45^+^ immune cells. **(C)** IL-17A was mainly derived from T lymphocytes rather than other lymphocytes. **(D)** IL-17A was mainly derived from CD8^+^ T cells comparing with other T cells, such as CD4^+^, CD3^+^CD4^−^CD8^−^ (DN), γδT, and NKT. **(E)** The expession of Tc17 in Control (*n* = 6), CRSsNP (*n* = 17), and CRSwNP (*n* = 48). (**F**) The expession of Th17 in Control (*n* = 6), CRSsNP (*n* = 17), and CRSwNP (*n* = 47). ^*^P < 0.05; ^**^P < 0.01; ^***^P < 0.001; ns, *P* > 0.05.

Having shown that patients with CRSwNP had increased IL-17A expression, a separate set of experiments was performed to determine the possible cell source(s) of IL-17A in CRSwNP tissues. Flow cytometric analysis after stimulation with PMA/ionomycin revealed that IL-17A was mainly located in lymphocytes but not monocytes, eosinophils, neutrophils (*P* < 0.001) (Figure 2A). Further analysis showed that IL-17A was predominately majority produced by CD45^+^ immune cells (*P* < 0.001) (Figure 2B). Then gating on CD3 to further analyze lymphocyte subtypes, which showed that IL-17A was almost exclusively produced by CD3^+^ T cells compared with B cells, NK cells and other CD3^−^ cells (*P* < 0.001) (Figure 2C). Different subtypes of T cells were next analyzed and although Tc cells (CD3^+^CD8^+^) were found to be the major cellular source of IL-17A (45.85 ± 7.21%), a lesser degree of IL-17A expression was found in CD4^+^ T helper cells (Th cells, CD3^+^CD4^+^, 30.25 ± 6.56%) and DN (double negative, CD3^+^CD4^−^CD8^−^, 21.60 ± 5.41%), as well as small amounts in γδT (4.68 ± 1.76%), and NKT (1.73 ± 0.81%) (Figure 2D). To further characterize IL-17A expression, the percentages of IL-17A^+^CD8^+^ T cells and IL-17A^+^CD4^+^ T cells were quantified in patients with CRSsNP, CRSwNP, and control subjects. Compared with those in controls, increased frequencies of Tc17 and Th17 in NMCs were found in CRSwNP (*P* = 0.005, *P* = 0.027) (Figures 2E,F). Tc17 was markedly higher in CRSwNP than CRSsNP (*P* = 0.023) (Figure 2E).

### Differential expression of IL-17A in nasal tissues and peripheral blood

**Figure 3:**
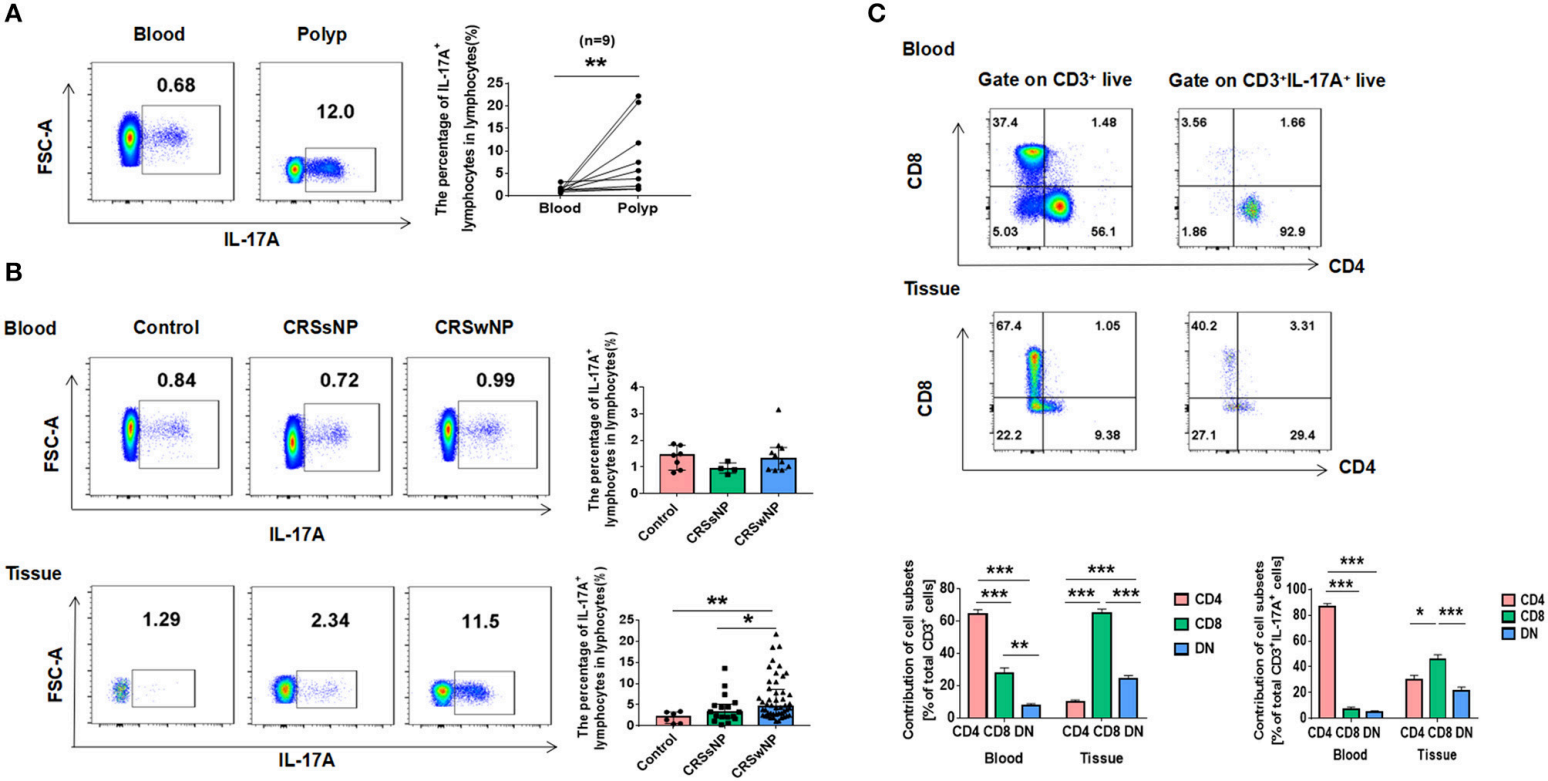
Differential expressions of IL-17A in nasal tissues and peripheral blood cells. **(A)** Flow cytometric analysis of the percentage of IL-17A^+^ lymphocytes in lymphocytes from polyp and paired peripheral blood cells. **(B)** The percentage of IL-17A^+^ lymphocytes in lymphocytes in Control, CRSsNP and CRSwNP derived from peripheral blood and nasal tissues (blood: Control = 7, CRSsNP = 4, CRSwNP = 10; tissues: Control = 6, CRSsNP = 17, CRSwNP = 48). **(C)** Expression of T-cells phenotypic markers and T-cell subets contribution to IL-17A production derived from blood and polyps (blood: *n* = 10, tissue: *n* = 32). ^*^P < 0.05; ^**^P < 0.01; ^***^P < 0.001; ns, *P* > 0.05.

In order to determine if the increased expression of IL-17A in CRSwNP could possibly due to local inflammation of the nasal mucosa or systemic inflammatory response, differences in IL-17A expression by flow cytometry were compared between blood and polyps from CRSwNP patients. On the paired comparison of IL-17A^+^ lymphocytes between blood and polyps from the same subjects, IL-17A^+^ lymphocyte percentages derived from polyps showed significantly higher IL-17A^+^ expression in contrast to negligible IL-17A expression in peripheral blood specimens from the same donors (*P* = 0.008) (Figure 3A). To further confirm these findings, the percentage of IL-17A^+^ lymphocytes in lymphocytes was compared to that observed in control, CRSsNP and CRSwNP derived from peripheral blood and nasal tissues. The data showed a markedly higher percentage of IL-17A^+^ lymphocytes from nasal tissues in patients with CRSwNP compared to that found in control and CRSsNP specimens (*P* = 0.009, *P* = 0.049) and no difference from that in peripheral blood (Figure 3B). An additional set of studies were performed to determine the T cell immunotype distributions between blood and polyps from CRSwNP patients. The results revealed that CD8^+^ T cells accounted for most of T cells in the nasal tissues while CD4^+^ T cells accounted for most of the peripheral blood. Tc cells (CD3^+^CD8^+^) are major IL-17A producers in NMCs from CRSwNP, Th cells (CD3^+^CD4^+^) are almost exclusively IL-17A producers in peripheral blood (Figure 3C).

### The expression of MMPs in CRS

**Figure 4:**
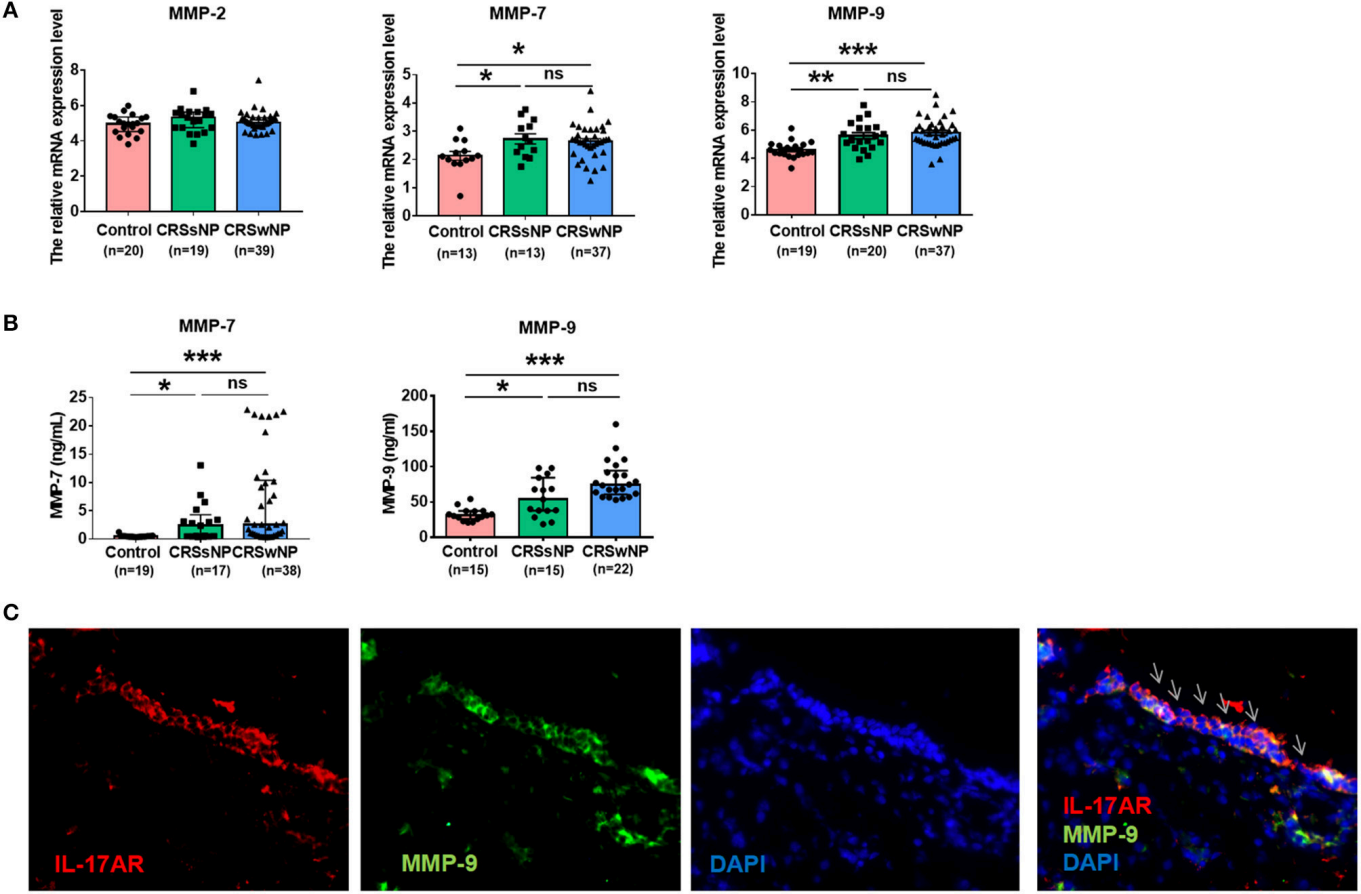
The expression of metalloproteinases (MMPs) and IL-17AR in CRS. **(A)** The mRNA expression levels of MMP-2, MMP-7, and MMP-9 were expressed relative to the expression level of β2 microglobulin (β2M) reference gene. **(B)** The protein expression levels of MMP-7 and MMP-9 were assayed by ELISA. **(C)** The co-expression of IL-17AR and MMP-9 in the nasal tissues from the CRSwNP patients. IL-17AR, MMP-9, and Nuclei were stained in red, green, and blue, respectively by Immunofluorescence. Arrows show IL-17AR/MMP-9 double-positive cells (magnification, 400 ×). ^*^P < 0.05; ^**^P < 0.01; ^***^P < 0.001; ns, *P* > 0.05.

To investigate whether MMPs might play a role in CRS, the mRNA expression of MMP family members including MMP-2, MMP-7, and MMP-9 in patients with CRSwNP was compared with that seen in controls. There was no difference in MMP-2 mRNA expression between them. MMP-7 mRNA expression was comparably upregulated in CRSwNP and CRSsNP compared with controls (*P* = 0.011, *P* = 0.02). And MMP-9 mRNA expression was significantly increased in CRSwNP and CRSsNP in comparison with controls (*P* < 0.001, *P* = 0.001). MMP-9 mRNA expression was higher in CRSwNP than CRSsNP, but the difference was not significant (Figure 4A). Further analysis of the protein expression of MMP-7 and MMP-9, we obtained tissue homogenates from patients with CRSsNP, CRSwNP, and control subjects, ELISA showed that the protein expression of MMP-7 and MMP-9 was in line with gene expression levels (Figure 4B).

### The co-expression of IL-17AR and MMP-9 in CRSwNP

To quantify the degree of IL-17AR and MMP-9 co-expression and their cellular locations, we first performed immunofluorescence staining with antibodies against IL-17AR to define the cell types expressing IL-17AR and then assess the relation between IL-17AR and MMP-9. We observed that IL-17AR co-localized with MMP-9 and they were mainly expressed in the epithelial cells in CRSwNP samples (Figure 4C).

### IL-17A resulted in increased production of MMP-9 in PHNECs and RPMI 2650

**Figure 5:**
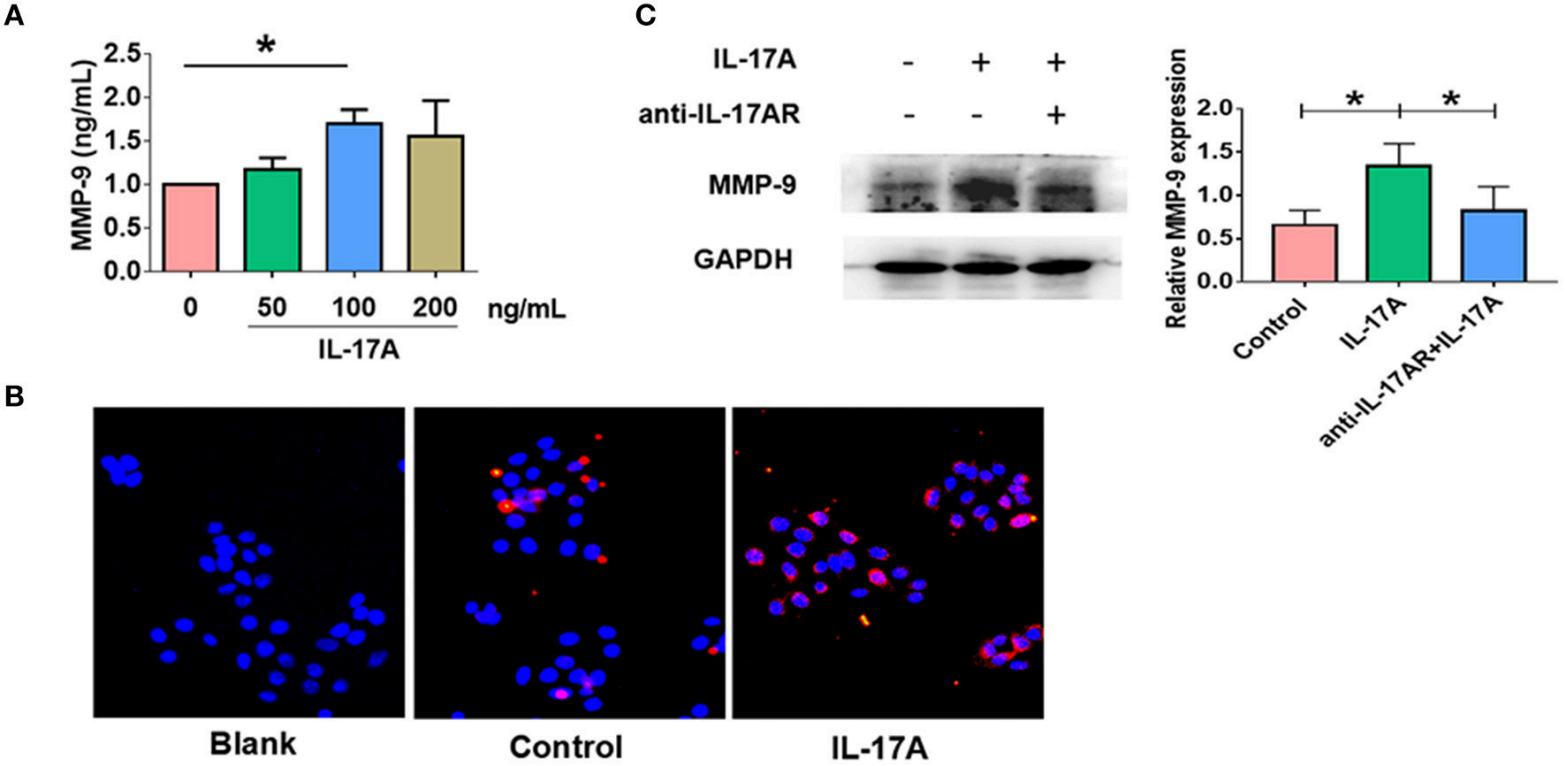
IL-17A up-regulated MMP-9 expression in in primary human nasal epithelial cells (PHNECs) and human nasal epithelial cell line (RPMI 2650). **(A)** PHNECs were incubated in various concentrations of IL-17A (0, 50, 100, 200 ng/mL) for 24 h. Concentration of MMP-9 in the supernatants was assayed by ELISA (*n* = 4). **(B)** RPMI 2650 cells were incubated with or without IL-17A (100 ng/mL) for 24 h. Immunofluorescence was performed to measure the expression of MMP-9 protein levels. **(C)** RPMI 2650 cells were incubated with IL-17A (100 ng/mL) and anti-IL-17AR for 24 h. Concentration of MMP-9 protein levels were assayed by WB (*n* = 3). ^*^P < 0.05.

Given IL-17AR and MMP-9 co-localized, we next asked whether IL-17A could promote MMP-9 expression in nasal epithelial cells. PHNECs were treated with gradient concentration of IL-17A for 24 h. ELISA showed that the protein expression of MMP-9 was significantly higher in response to IL-17A with an optimal concentration of 100 ng/ml (*P* = 0.024) (Figure 5A). For further verification, RPMI 2650 was stimulated with IL-17A (100 ng/ml) for 24 h. Immunofluorescence showed an increasing MMP-9 expression in epithelial cells incubated with IL-17A (Figure 5B). IL-17AR co-localization with MMP-9 was next examined and was found to be mainly expressed in the epithelial cells in CRSwNP samples (Figure 4B). In order to confirm that IL-17A-induced MMP-9 production *via* IL-17AR signaling, RPMI 2650 was pretreated with anti-IL-17AR for 1h before IL-17A treatment. As expected, the production of IL-17A induced MMP-9 (*P* = 0.022) was significantly decreased in anti-IL-17AR combined with IL-17A treatment compared with only IL-17A treatment (*P* = 0.03) (Figure 5C). In addition, the results also indirectly confirmed the effect of IL-17A on MMP-9. Collectively, our data showed that IL-17A could up-regulate the MMP-9 expression in both PHNECs and RPMI 2650. In contrast, there were no changes in the expression of MMP-7 in response to IL-17A (Supplement Figure 1).

### IL-17A-induced MMP-9 production in PHNECs and RPMI 2650 via NF-κB signaling

**Figure 6:**
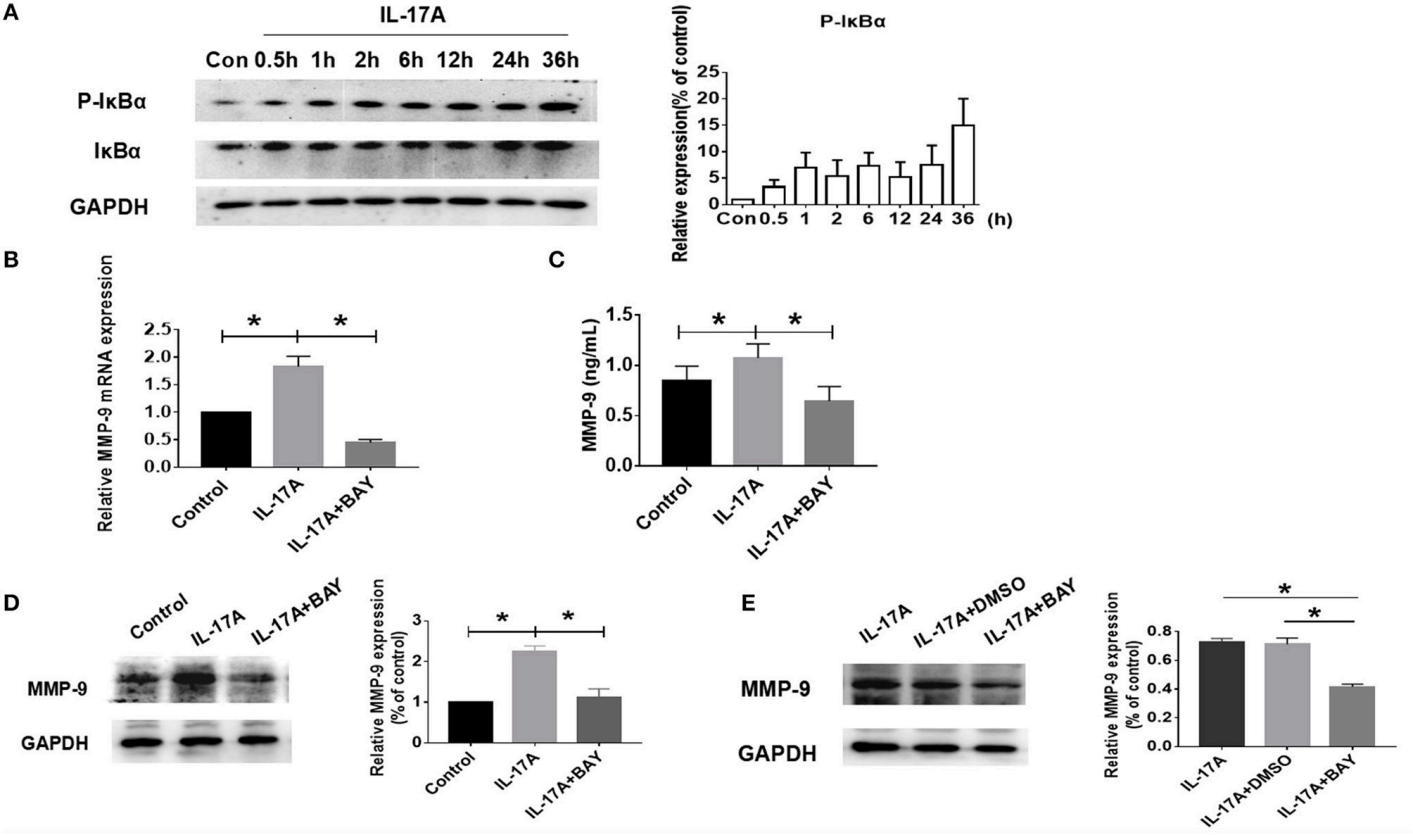
IL-17A up-regulated MMP-9 production in PHNECs and RPMI 2650 via NF-κB pathway. **(A)** RPMI 2650 cells were incubated with 100 ng/ml IL-17A in various times. Western blot was performed to analyze the expression of NF-κB protein levels (*n* = 3). **(B,C)** Primary HNECs were incubated with or without IL-17A (100 ng/mL) + BAY (5 μM) for 24 h. QRT-PCR (*n* = 3) **(B)** and ELISA (*n* = 4) **(C)** were performed to measure the expression of MMP-9 transcript levels and protein levels. **(D)** RPMI 2650 cells was incubated with or without IL-17A (100 ng/mL) + BAY (5 μM) for 24 h. WB was performed to measure the expression of MMP-9 protein levels (*n* = 3). **(E)** RPMI 2650 was incubated with IL-17A (100 ng/mL) + BAY (5 μM) /DMSO for 24 h. WB was performed to measure the expression of MMP-9 protein levels (*n* = 3). ^*^P < 0.05.

Since the mechanism of action of IL-17A has been reported to occur by activation of the nuclear factor kappa-light-chain-enhancer of activated B cells (NF-κB) (39, 40), the role of NF-κB was next examined in IL-17A-induced MMP-9 production. Western Blot was used to measure the expression and phosphorylation levels of IκBα proteins in RPMI 2650 after treatment with 100 ng/ml IL-17A at various time intervals. The results revealed enhanced phosphorylation of IκBα was present as early as 30 min and continued (Figure 6A). IL-17A rapidly activated NF-κB signaling. BAY 11-7082, a NF-κB small molecule inhibitor, was employed to confirm the role of the signaling pathway in IL-17A-induced MMP-9 production. The concentration of 5 μM of BAY 11-7082 used was found to be optimal and the first proof to effectively inhibit NF-κB signaling (Supplement Figure 2). PHNECs were pretreated with BAY 11-7082 for 1 h before IL-17A treatment, finally both the production of MMP-9 mRNA and protein were attenuated significantly by PCR (*P* = 0.029) and ELISA (*P* = 0.015), respectively (Figures 6B,C). This same phenomenon also seen in RPMI 2650 by WB (*P* = 0.025) (Figure 6D). Since BAY 11-7082 is dissolved in DMSO, in order to exclude the effects of non-specific interference, we treated RPMI 2650 with DMSO. Compared with BAY 11-7082, DMSO did not reduce the IL-17A-mediated the increasing of MMP-9 (Figure 6E). Collectively, these data indicate that IL-17A promotes MMP-9 expression in PHNECs and RPMI 2650 *via* NF-κB signaling.

## Discussion

The focus of the present study on molecular mechanisms contributing to the pathogenesis of chronic rhinosinusitis (CRS) derives both from its clinical complexity with protean clinical manifestations as well its pathogenetic heterogeneity with involvement of multiple inflammatory factors. Consistent with previous studies (33, 41, 42), our results confirmed that IL-17A protein is highly expressed in sinonasal biopsies from CRSwNP compared with those from CRSsNP and controls. Dissimilar to other studies, however, is our use of flow cytometry in the analysis of the pathogenetic role of IL-17A^+^ live cells. This not only permitted the quantitative measurement of IL-17A producing cells in nasal tissue of patients with CRS which showed an increased IL-17A^+^ live cells in both CRSwNP and CRSsNP from controls but also that CRSwNP exhibited higher IL-17A^+^ live cells than CRSsNP. Collectively, our results confirmed and extended the findings of others that increasing IL-17A expression plays a key pathogenetic role in CRSwNP.

The identification of the cellular source of IL-17A in CRSwNP fulfilled the first goal of the present study. Although it is generally known that both adaptive immune T cells and innate cell populations can produce IL-17, a pro-inflammatory cytokine that plays a key role in mucosal tissues (43), the specific cellular source of this cytokine has been less well identified. By careful investigative dissection of lymphocytes from other cells, including monocytes, eosinophils, neutrophils, IL-17A was found to be mainly derived from lymphocytes. When gating on CD45, we observed that IL-17A was predominately produced by CD45^+^ immune cells. Further analysis of lymphocyte subtypes showed that IL-17A was almost exclusively produced by CD3^+^ T cells rather than B cells, NK cells, and other CD3^−^ cells. Based upon and consistent with the findings of Molet et al. who reported that the main IL-17^+^ inflammatory cell type in American patients with nasal polyps was the T cell (44), we further analyzed different subtypes of T cells, and unexpectedly observed for the first time that Tc cells (CD3^+^CD8^+^) are the major cellular source of IL-17A although a reduced degree of production was also observed in Th cells (CD3^+^CD4^+^) and DN (double negative, CD3^+^CD4^−^CD8^−^), as well as smaller amounts in γδT cells and NKT cells. These results, however, differ from the findings of Ma et al. which demonstrated that the frequency of nasal CD8^+^ T cells producing IL-17A was less than or comparable to that of CD4^+^ T cells producing IL-17A in IL-17A-positive CD3^+^ T cells (45). These discrepant results may be related to methodologic differences in measurement of CD4^+^ T cells. In the studies of Ma et al., the labeling technique for CD4^+^ T cells employed defined these cells as CD3^+^CD8^−^ T cells, differing from CD3^+^CD4^+^ T cells identified in our study. This focus on CD3^+^CD8^−^ T cells would mistakenly assign non-CD4^+^ T cells into CD4, such as double negative T-cells (DN, CD3^+^CD4^−^CD8^−^) (46).

In the present study, DN (double negative) T cells, a small CD3 cell population lacking both CD4 and CD8, were shown to also produce IL-17A, a finding that supports the idea that this T-cell subset may be actively involved in the immunopathogenesis of CRSwNP. However, DN T cells are poorly understood and have been largely ignored by immunologists (47). γδT cells which were thought to be another important source of IL-17A, also play an important role in promoting inflammation and regulating immunity (48). Recently, γδT cells have been proposed to be early producers of IL-17 following tissue injury or exposure to pathogens (49). In the present study, IL-17-secreting γδT cells were identified, but only in small quantities. The reason for the paucity of these cells is not clear but may have been related to the timing of specimen collection which have missed the optimal point detection in the acute inflammatory response. Natural killer T cells (NKT) are innate-like cells which co-express cell-surface markers that are encoded by the natural killer (NK) locus, [e.g., NK1.1 (mouse) or CD56^+^ (human)] as well as an antigen receptor (TCR) characteristic of conventional T cells (50). IL-17-producing NKT cells are part of innate IL-17 producers and play a key role in immune surveillance (43). IL-17-secreting NKT cells are thought to belong to a distinct lineage that develops in the thymus, which should be called “natural NKT17” cells, in contrast with the “induced NKT17” cells generated in the periphery (51). In the present study, although only a small amount of IL-17A production was observed in NKT cells, the results revealed it is possible to induce secretion of IL-17A from NKT cells outside the thymus under the internal environment of CRS. It is of great value to further explore the secretion of IL-17A by induced NKT cells responding to some particular cytokines.

In most inflammatory diseases, T helper 17 (Th17) cells have been found to be the primary source of IL-17A and its homologous family member, IL-17 (17, 52). Most investigations have concentrated on IL-17A produced by a specific subset of CD4^+^ T cells (Th17). In recent years, studies have also focused on Tc17 cells, a subset of IL-17-secreting CD8 T cells that were formally identified in 2009 as a novel subtype of cytotoxic T cells (53) which have been identified in several human inflammatory diseases, such as psoriatic (54), chronic hepatitis C virus (HCV) infection (55). However, unlike Th17 cells, Tc17 cells have received only marginal attention. Interestingly, Tc17 was identified to be highly expressed in nasal polyps in the present study, a finding that may provide a disease model for further study.

In the present study, the unique features of inflammation in the nasal mucosa of patients with CRSwNP were compared to other biomarkers seen in other inflammatory diseases. It would seem logical to have identified an accumulation of CD8^+^ T cells in polyps or nasal mucosa of CRSwNP, with a relative deficiency of CD4^+^ T cells, which would be consistent with some studies that report that CD8^+^ T cells were the predominant inflammatory cells in CRSwNP (56, 57). However, the majority of most previous studies focus on the role of CD4^+^ T in other chronic inflammatory disorders (58). In addition, different cellular patterns of inflammatory cellular infiltration between blood and polyp from CRSwNP were compared The results revealed that CD8^+^ T cells accounted for most of T cells in the nasal tissue while CD4^+^ T cells accounted for most of the T cell composition in peripheral blood. Based on this tissue/blood dichotomy, it is reasonable to conclude that IL-17 may be involved in local immune responses in CRSwNPs in agreement with the findings of Jiang et al. (41).

In this study, our data not only showed the markedly higher percentage of IL-17A^+^ lymphocytes only be seen in the polyps, not blood from CRSwNP compared with controls, but also found that IL-17A^+^ lymphocytes derived from polyps expressed significantly higher percentages, whereas negligible IL-17A expression in peripheral blood from the same donors. Besides, the major IL-17A producers in polpys and blood were Tc cells and Th cells respectively. Therefore, CRS inflammation is mainly confined to the mucosal compartment, rather than a reflection of a systemic immune disorder. This may have a guiding effect on topical treatment.

A second goal of the present study was to examine the relationship between of IL-17A and the tissue remodeling in CRSwNP. Current studies have demonstrated that IL-17 is associated with neutrophil recruitment and survival in nasal polyp (59, 60). Other studies have reported that IL-17 can increase the production of proinflammatory cytokines, chemokines, and MMPs from various tissues and cell types (29). Since that MMPs, a family of Ca^2+^-activated, zinc-dependent endopeptidases, play a key role in the degradation of basement membranes and extracellular matrix (ECM) and contribute to edema formation (7), the relationship between IL-17 and MMPs seems worthy of study. MMP-2 and MMP-9 are gelatinases and are the vital members of MMPs family. MMP-7, called Matrilysin-1, is a small molecule with a wide range of substrates. Since these molecules are involved in the metabolism of type IV collagen in the basement membrane, their possible pathogenetic role has attracted considerable attention in CRS tissue remodeling. TIMPs are the most important inhibitors of MMPs *in vivo*, in which TIMP-1 plays a major role (61). Precious study revealed that MMPs are important potential therapeutic targets for CRS. Tetracycline and macrocyclic lipid drugs (MMP inhibitors), which can reduce the formation of nasal polyps through down-regulation of MMP-9 levels (62). In the present study, we confirmed that the gene and protein expression levels of MMP-7 and MMP-9 significantly increased in CRSwNP in comparison with CRSsNP and controls, it was in agreement with other reports (10, 11). But there was no difference in MMP-2 expression among them. Although there were no significant differences between the concentrations of MMP-7 and MMP-9 in patients with CRSwNP and CRSsNP, CRSwNP expressed relative lack of TIMP-1 expression vs. CRSsNP in our previous research (63). The imbalance between MMPs and their tissue inhibitor of metalloproteinases (TIMPs) is one of the most leading causes of pathological tissue remodeling in CRS (9, 10, 11). Since IL-17A has been reported to promote the invasion of inflammatory cells into cancer cells via upregulating the expression of MMP-2 and MMP-9 (30, 31), it seemed reasonable to hypothesize that IL-17A could promote MMPs expression in human nasal epithelium cells. In this study, we found IL-17AR co-localized with MMP-9 and they were mainly expressed in the epithelial cells in CRSwNP samples. To validate our hypothesis, cultured nasal epithelial cells were isolated from nasal polyps (PHNECs) and the epithelial cells line, RPMI 2650 (35), pretreated these two kinds of cells with human IL-17A and/or IL-17AR neutralization. IL-17A treatment was found to up-regulate MMP-9 expression, with an optimal stimulatory concentration of 100 ng/mL, and this up-regulation could be eliminated by IL-17AR pretreated. This indicates that IL-17A-induced MMP-9 production via binding to IL-17AR to activate downstream signaling. In contrast, there were no changes in the expression of MMP-7 in response to IL-17A (Supplement Figure 1).

NF-κB transcription binding sites located in human MMP-9 promoter region and revealed that NF-κB is the key point in the upregulation of MMP-9 expression (64). Consistently, previous studies have shown that MMP-9 is a downstream gene in the NF-κB pathway (65, 66). Additionally, NF-κB has been reported as a downstream target of IL-17A signaling pathway (39, 40). So we suspect that NF-κB may participate in the mechanism of IL-17A regulating MMP-9. In the present study, we confirmed that IL-17A could activate the NF-κB pathway. Furthermore, PHNECs and RPMI 2650 were pretreated with BAY 11-7082 (IκB phosphorylation inhibitor) (67) combined with IL-17A or not. Different concentration gradients of BAY11-7082 were studied to obtain BAY's optimal inhibitory concentration of 5 μM (Supplement Figure 2). It was confirmed that the upregulated MMP9 expression induced by IL-17A could be abrogated by NF-κB inhibition, indicating that IL-17A could induce MMP-9 production in nasal epithelial cells via activating the NF-κB pathway.

In summary, the results of the present study suggested that the local inflammatory milieu in CRSwNP may induce IL-17A expression. Tc cells (CD3^+^CD8^+^) are major IL-17A producers. IL-17A up-regulates the expression of MMP-9 via NF-κB pathway in nasal epithelial cells, suggesting that IL-17A might play a crucial role in tissue remodeling of CRSwNP.

## Author contributions

XC, LC, and XL contributed equally as co-first author. GZ and SZ designed the research and charge correspondence. XC, LC, and XL performed the experiments. JH, LY, XPL, and JZ helped carrying out experiments. XC, ZH, ZW, and XW analyzed the data. XC, LC, GZ, and SZ wrote the manuscript. JB edited manuscript. All the authors read and approved the final manuscript.

### Conflict of interest statement

The authors declare that the research was conducted in the absence of any commercial or financial relationships that could be construed as a potential conflict of interest.
