# Supplementary material for: Tc17/IL-17A Up-Regulated the Expression of MMP-9 via NF-κB Pathway in Nasal Epithelial Cells of Patients With Chronic Rhinosinusitis
Source: Front Immunol. 2018 Sep 19;9:2121. doi: 10.3389/fimmu.2018.02121 (PMC6156140; doi:10.3389/fimmu.2018.02121)
Supplement: Supplementary file 1 [file Data_Sheet_1.PDF]

Supplementary Material

Tc17/IL-17A up-regulated the expression of MMP-9 via NF-κB pathway in nasal epithelial cells of patients with chronic rhinosinusitis.

Xiaohong Chen*, Lihong Chang, Xia Li
Correspondence: Gehua Zhang, gehuazh@hotmail.com
Song Guo Zheng, szheng1@pennstatehealth.psu.edu

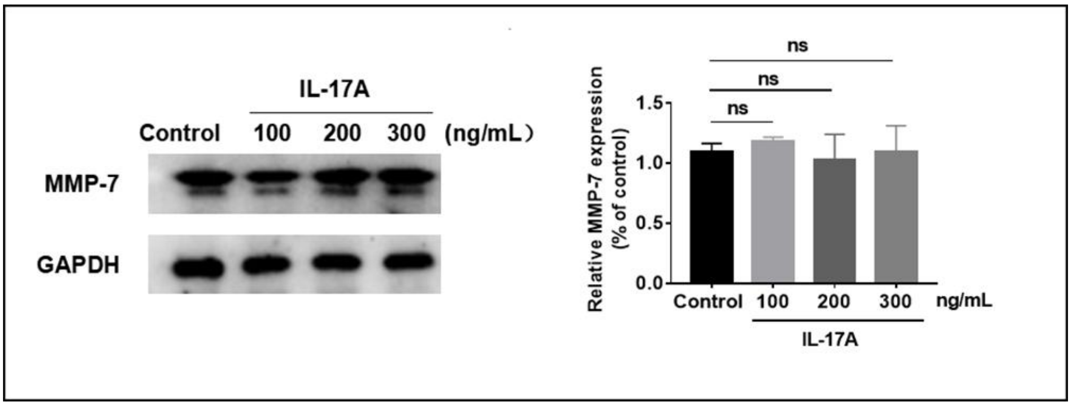

Supplement Figure 1. IL-17A didn't upregulate MMP-7 expression in human nasal epithelial cells (HNECs). PHNECs were incubated in various concentrations of IL-17A (0, 100, 200, 300 ng/mL) for 24 h. Concentration of MMP-7 protein levels were assayed by WB. *$P < 0.05$; **$P < 0.01$; ***$P < 0.001$; NS, $P > 0.05$.

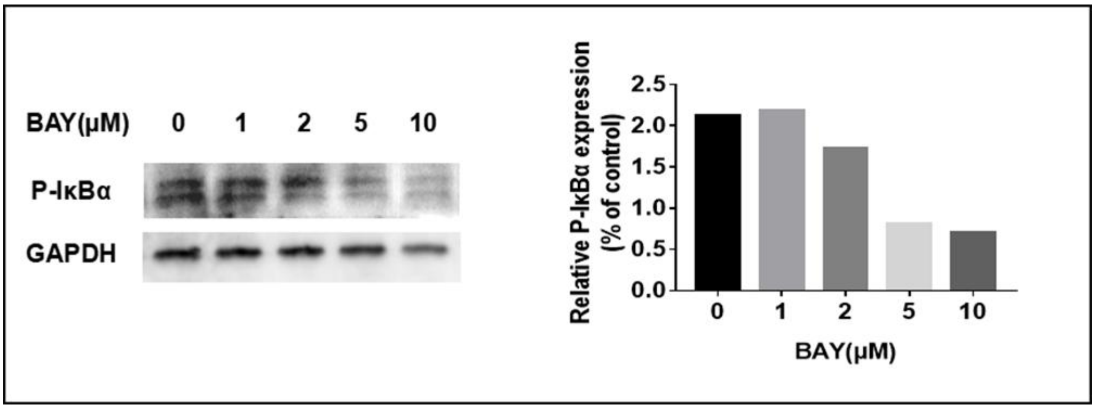

Supplement Figure 2. BAY 11-7082 down-regulated P-IκBα production in human nasal epithelial cell line (RPMI 2650). RPMI 2650 were incubated in various concentrations of BAY 11-7082 (0, 1, 2, 5, 10μM) for 24 h. Concentration of P-IκBα protein levels were assayed by WB.
